# Methylene Blue Application to Lessen Pain: Its Analgesic Effect and Mechanism

**DOI:** 10.3389/fnins.2021.663650

**Published:** 2021-05-17

**Authors:** Seung Won Lee, Hee Chul Han

**Affiliations:** ^1^Good Doctor Research Institute, College of Medicine, Korea University, Seoul, South Korea; ^2^Department of Physiology, College of Medicine and Neuroscience Research Institute, Korea University, Seoul, South Korea

**Keywords:** methylene blue, pain, anti-inflammation, sodium current, denervation

## Abstract

Methylene blue (MB) is a cationic thiazine dye, widely used as a biological stain and chemical indicator. Growing evidence have revealed that MB functions to restore abnormal vasodilation and notably it is implicated even in pain relief. Physicians began to inject MB into degenerated disks to relieve pain in patients with chronic discogenic low back pain (CDLBP), and some of them achieved remarkable outcomes. For osteoarthritis and colitis, MB abates inflammation by suppressing nitric oxide production, and ultimately relieves pain. However, despite this clinical efficacy, MB has not attracted much public attention in terms of pain relief. Accordingly, this review focuses on how MB lessens pain, noting three major actions of this dye: anti-inflammation, sodium current reduction, and denervation. Moreover, we showed controversies over the efficacy of MB on CDLBP and raised also toxicity issues to look into the limitation of MB application. This analysis is the first attempt to illustrate its analgesic effects, which may offer a novel insight into MB as a pain-relief dye.

## Introduction

In 1876, German chemist Heinrich Caro synthesized methylene blue (MB) for the first time in history, which was basically applied for textiles as an aniline dye. Around the same time, it was found that MB is capable of staining cells by binding to their structures, in addition, sometimes inactivating bacteria. This discovery prepared the innovative ground for biological or medical studies related to MB. Numerous scientists applied it to a variety of animal and bacterial studies, importantly Paul Ehrlich introduced it to humans in 1891 as an anti-malarial agent. Indeed, this dye has been introduced to treat different diseases even including dementia, cancer, and depression (Wainwright and Crossley, 2002; Schirmer et al., 2003; Schirmer et al., 2011).

In the present day, MB is primarily known for a vasoconstrictor. It downregulates basically nitric oxide (NO), which is responsible for relaxing vascular smooth muscle, and leads to vasoconstriction (Wolin et al., 1990; Pan et al., 2019). However, under pathological conditions, NO is overexpressed and then contributes to inflammation as a pro-inflammatory mediator (Luo and Cizkova, 2000; Lundberg et al., 2008; Leiper and Nandi, 2011). Of note, MB suppresses the iNOS/NO-mediated inflammatory signaling by directly downregulating inducible NO synthase (iNOS) (Cohen et al., 2000). In addition, P2X receptor family, long non-coding RNA (lncRNA), and inflammasome are also involved in MB-mediated anti-inflammation (Ahn et al., 2017; Li et al., 2018; Zheng and Li, 2019). Accordingly, MB application can be an important strategy to reduce inflammation and pain.

In general, voltage-gated sodium channels (VGSCs) play an important role in evoking action potentials (APs) and, when activated, they consequently contribute to exciting neurons and thus facilitating communication with other ones. Interestingly, MB decreased significantly *I*_*NA*_ (voltage-gated sodium currents) in hippocampal CA1 neurons and, more importantly, attenuated markedly neural firing rates in the afferent nerve fibers (Zhang et al., 2010; Lee et al., 2021), which implies that MB may impede pain transmission by dampening neuronal excitability elicited by VGSCs.

In addition, MB may contribute to pain reduction by hindering or damaging nerve connection to tissues, which is referred to as denervation. Indeed, it can make affected nerve fibers or neurons incapable of sensing pain. Peng et al. (2007) conducted intradiscal MB injection in patients with chronic discogenic low back pain (CDLBP) to relieve pain for the first time. Most of patients showed encouraging results and this improvement lasted at least one year. Moreover, in a case, there were no noticeable side effects and complications in those patients even after prolonged follow-ups (Peng et al., 2010). However, as opposed to expectations, these outstanding outcomes faced a lot of challenges. We will deal with the controversies around the results in the relevant section.

Despite these remarkable reports, MB has not drawn much attention from the public specifically concerning pain. Thus, in this review, we will show MB-driven analgesic effects and their possible mechanisms along with the relevant experimental evidence and clinical cases. Finally, we will provide a novel insight into MB as a pain reliever.

## MB and Anti-Inflammation

### Blockade of NOS/NO-Mediated Inflammatory Signaling

**FIGURE 1:**
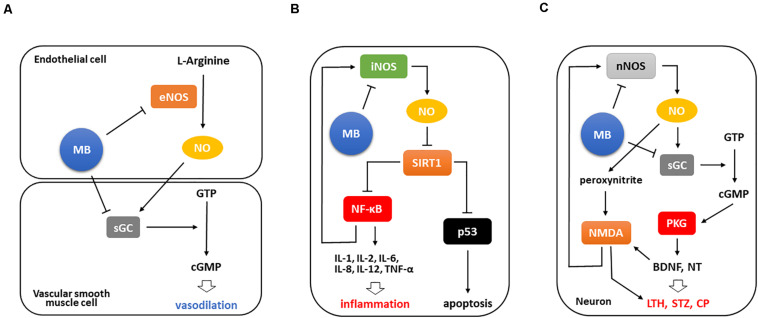
MB is involved in anti-inflammation by suppressing iNOS/NO-NF-κB pathway. **(A)** Basically, MB downregulates both eNOS and sGC that are major factors converting GTP to cGMP, ultimately leading to vasoconstriction. **(B)** Upon tissue injury, iNOS functions as a strong inflammatory mediator in different types of cells. It inhibits Sirt1 activation by NO-mediated S-nitrosylation, which in turn activates NF-κB and p53 to facilitate inflammatory cytokine expression and apoptosis, respectively. Of note, NF-κB activation intensifies these events by activating iNOS/NO-NF-κB pathway. Conversely, MB directly abates iNOS expression and moreover decreases the binding of NF-κB to iNOS promoter, which consequently interrupts this inflammatory signaling. **(C)** Meanwhile, NMDA receptors are activated during nerve injury and induces Ca^2+^ influx, which then results in the excessive expression of nNOS and markedly activates nNOS/NO signaling. The increased NO production stimulates NMDA receptors and triggers NO/cGMP/PKG cascade, which promotes the subsequent BNDF upregulation and neurotransmitter release and ultimately induces long-term hyperexcitability and central sensitization. Notably, BDNF and peroxynitrite potentiate NMDA receptors, which stimulate nNOS expression again. However, MB weakens these responses by inhibiting nNOS and sGC activation, thus may prevent the development of persistent pain.LTH, long-term hyperexcitability; STZ, sensitization; NT, neurotransmitter; CP, chronic pain.

Inflammation is tightly correlated with pain as a critical cause. It directly or indirectly induces nociceptive responses and in many cases underlies pain or pain-related diseases (Zhang H. et al., 2016; Harth and Nielson, 2019; Matsuda et al., 2019). It is well known that MB decreases NO formation by directly suppressing endothelial NOS (eNOS) expression, and blocks also the conversion of guanosine triphosphate (GTP) to cyclic guanosine monophosphate (cGMP) by suppressing soluble guanylate cyclase (sGC) expression in vascular smooth muscles, which in turn leads to vasoconstriction (Figure 1A; Wolin et al., 1990; Pan et al., 2019).

### The MB-Induced Interruption of iNOS/NO-NF-κB Signaling

Based on this pathway, MB weakens inflammation since NO plays a crucial role in the pathogenesis of inflammation as a pro-inflammatory mediator. This dye inhibits iNOS/NO pathway to affect nuclear factor kappa-light-chain-enhancer of activated B cells (NF-κB) activation. Silent information regulator 1 (Sirt1), which is a deacetylase and have a protective role under stress conditions, inactivates NF-κB and tumor protein p53 (p53) by deacetylating them. In contrast, iNOS/NO activates these two transcription factors by inactivating Sirt1 via S-nitrosylation (Nakazawa et al., 2017). NF-κB activation facilitates inflammatory cytokine expression, importantly, upregulates iNOS to enhance inflammatory signaling (Nakazawa et al., 2017; Jiang et al., 2020), and p53 activation induces cell death (Aubrey et al., 2018). However, MB prevents these events by downregulating iNOS in multiple inflammatory milieus. It directly inhibits iNOS expression and also attenuates the binding of NF-κB to iNOS promoter, thus resulting in the blockade of iNOS/NO-NF-κB signaling (Figure 1B; Huang et al., 2015).

Specifically, in human cartilage explants, MB decreased NO accumulation and iNOS expression in a dose dependent manner, and upregulated transforming growth factor beta (TGF-β) receptors, known as an important factor for cartilage matrix synthesis. As a result, it prevented the degradation of cartilage matrix and proteoglycan (Cohen et al., 2000). In ulcerative colitis rats, MB decreased remarkably NO production and inflammatory cytokine (IL-1β, IL-6, and TNF-α) levels. Functionally, with the alleviated tissue injury and edema in the submucosa, there was a significant improvement in intestinal permeability after MB application (Dinc et al., 2015). Accordingly, these studies demonstrated that MB has a critical role in negatively modulating NOS expression and NO production and ultimately is able to induce functional improvement in the inflamed tissues.

During nerve injury, Toll-like receptor 4 (TLR4) initiates neuroimmune activation in the nervous system. Notably, TLR4 activates NF-κB by mediating its nuclear localization, which in turn promotes pro-inflammatory cytokine expression and ultimately leads to inflammatory hyperalgesia (Lacagnina et al., 2018; Yadav and Surolia, 2019). Tanga et al. demonstrated for the first time that upon nerve injury (L5 nerve transection), behavioral hypersensitivity is induced via TLR4/NF-κB pathway, suggesting that TLR4 activation is a critical cause of the pathogenesis of neuropathic and chronic pain (Tanga et al., 2005). In this context, targeting TLR4/NF-κB pathway can be recommended as a decisive therapeutic strategy to relieve nerve injury–induced neuroinflammation and pain (Yadav and Surolia, 2019; Ye et al., 2020).

Interestingly, TLR4 is much involved in iNOS activation (Deng et al., 2015). This implies that MB may participate in suppressing the development and maintenance of neuropathic pain by downregulating directly iNOS and weakening NF-κB activation as previously described. A clinical study showed that systemic MB administration improved 10 patients with chronic refractory neuropathic pain, which was assumably due to the MB-mediated blockade of iNOS/NO pathway (Miclescu et al., 2015).

### The MB-Induced Interruption of nNOS/cGMP/PKG Signaling

Lastly, neuronal NOS (nNOS), predominantly found in neurons, is also responsible for NO production and engaged in NO-mediated pathway (Kourosh-Arami et al., 2020). This enzyme has been much investigated specifically concerning chronic and inflammatory pain. In particular, nNOS has a critical role in the development of chronic and neuropathic pain upon nerve injury (Kim et al., 2011), while iNOS is partially related to it (Rocha et al., 2020). Interestingly, a study demonstrated that spinal nNOS, not eNOS and iNOS, is significantly upregulated after inflammatory pain induction and contributes to central sensitization (Chu et al., 2005). In this respect, nNOS has been highlighted as a target to dampen pain in both inflammatory and non-inflammatory milieus (Mukherjee et al., 2014; Demir et al., 2019). Moreover, it was found that NO/cGMP-mediated pathway, a downstream target of nNOS, is crucial for the development and maintenance of pain (Li et al., 2020), and cGMP protein kinase (PKG) is considered as a potent generator in this event since leading to long-term hyperexcitability and pain sensitization in neurons by inducing neurotransmitter release and upregulating brain-derived neurotrophic factor (BDNF) (Lewin and Walters, 1999; Sung et al., 2017; Wang et al., 2021). Furthermore, BDNF released during nerve injury and peroxynitrite activated via nNOS/NO signaling potentiate N-methyl-D-aspartate (NMDA) receptors, which then induces long-term hyperexcitability and upregulates nNOS, respectively (Pall, 2002; Chen et al., 2014; Figure 1C).

Importantly, MB is able to downregulate both nNOS and sGC, resulting in the inhibition of nNOS/NO-mediated signaling. Rey-Funes et al. (2016) demonstrated that MB protects retinal damage in rats with ischemic proliferative retinopathy, which is associated with local inflammation in the ischemic retina. MB suppressed the expression levels of nNOS and proangiogenic factors such as matrix metalloproteinase (MMP)-2 and MMP-9 and upregulated pigment epithelium-derived factor (PEDF), ultimately leading to the reduction of inner retinal thickness, gliosis, and retinal angiogenesis.

### Inhibited NOS Activation Potentiates Opioidergic Effects

Opioids are substances with similar effects to those of morphine and used primarily for lessening pain. Basically, opioids act by binding to opioid receptors (ORs), which show analgesia by reducing cyclic adenosine monophosphate (cAMP) and causing the resultant decrease in the excitatory ion channels (Quirion et al., 2020; Sun et al., 2020; Kaski et al., 2021).

However, a number of issues concerning their addiction and adverse effects have been raised. In effect, morphine application may induce inflammatory cytokine expression such as IL-1β, IL-6, and TNF-α, ultimately contributing to inflammation and neurotoxic events. Importantly, it was found that reduced nNOS activation not only enhances the antinociception of morphine but also inhibits the morphine-induced neurotoxicity by downregulating inflammatory mediators (Machelska et al., 1997; Osmanlıoğlu et al., 2020). In this regard, MB may also contribute to these events as a potent NOS inhibitor. A clinical report showed that oral MB administration relieved oral mucositis-related intractable pain and more importantly reduced significantly morphine requirement in the relevant patients (Roldan et al., 2017).

There are some links between TLR4, OR, and cholecystokinin (CCK). CCK, an important neurohormone, plays a variety of roles in the nervous system and notably engaged in pain reduction mainly via CCK-B receptors (Roca-Lapirot et al., 2019; Keppel Hesselink, 2020). Interestingly, this peptide functions to inhibit OR activity and thus reduces the morphine-induced antinociception (Torres-López et al., 2007; Yang et al., 2018). Meanwhile, upon nerve injury, *Tlr4* genes are significantly expressed in the midbrain and medulla of CCK-B receptor knockout mice compared to those of wild type, implying that CCK is involved in innate immunity (Kõks et al., 2008). This result is supported by recent studies that CCK has a pivotal role in anti-inflammation by decreasing inflammatory mediators (Funakoshi et al., 2019; Saia et al., 2020). It has not yet been confirmed that MB is related to these links. However, it is believed that MB possibly shows a synergistic effect with morphine and CCK, thus enhancing anti-inflammatory and analgesic effects.

### Downregulation of P2XR and lncRNA

#### MB-Mediated P2XR Downregulation Induces Anti-inflammation and Pain Relief

Purinergic P2X receptor subtypes have been highlighted as a nociceptor that causes inflammation and pain. These receptors are characterized by ligand-gated ionotropic channels activated in response to the binding of adenosine 5′-triphosphate (ATP), exhibiting a non-selective cationic permeability such as K^+^, Na^+^, and Ca^2+^ that can contribute to opening voltage-gated channels on neurons by depolarizing membrane potential and activate a diversity of intracellular Ca^2+^-dependent signaling pathways (Surprenant and North, 2009; Schmid and Evans, 2019). In particular, P2 × 3 and P2 × 2/3 receptors among the subunits are distributed in the dorsal root ganglion (DRG) and the central terminal of primary afferent fibers, mainly small-diameter unmyelinated C-fibers, and play a critical role in pain transmission in the periphery (North, 2004; Bernier et al., 2018; Stephan et al., 2018). Interestingly, MB is highly correlated with this P2X receptor-mediated events.

Li et al. showed that MB alleviated inflammation and pain in osteoarthritis (OA) rabbits by negatively modulating P2 × 3 receptors. In this study, after intra-articular MB administration, weight distribution in the hind paw was significantly restored and the swelling ratio in the inflamed knees also markedly declined. Inflammatory cytokine levels (TNF-α, IL-1β, IL-6, and IL-8) were also remarkably reduced in the articular cartilage with a significant decrease in P2 × 3 receptor expression. Moreover, this event was reversed by P2 × 3 overexpression (Li et al., 2018).

#### Decreased lncRNA Contributes to Anti-inflammation

Meanwhile, lncRNA has been highlighted as a new player in gene regulation. Previous studies revealed that increased lncRNA is a critical cause of neuropathic pain, and interacts with P2XRs to initiate and maintain pain (Zhao et al., 2013; Li et al., 2017). Also, it is much implicated in inflammatory events (Chen et al., 2019).

Interestingly, MB may contribute to anti-inflammation and pain relief by downregulating this lncRNA. Zheng and Li demonstrated that MB negatively regulates a long non-coding RNA (lncRNA), specifically a chondrocyte inflammation–associated lncRNA (CILinc02), overexpressed in osteoarthritic cartilage tissues and cultured OA cells, and decreased inflammatory cytokine levels (IL-1, IL-6, and IL-17). Of note, MB suppressed even chondrocyte degradation. Conversely, CILinc02 overexpression resulted in inflammation and apoptosis (Zheng and Li, 2019).

### Inhibition of Inflammasome Formation and Activation

Inflammasome is an intracellular multiprotein complex that functions to process cytokine precursors into their mature forms and induce an inflammatory form of programmed cell death termed pyroptosis, which, ultimately, has a crucial role in providing host defense against harmful intruders (Matsuda et al., 2019).

Upon stimulation, inflammasome initiates the expression of cytosolic sensing proteins, called a priming step, which include nucleotide-binding oligomerization domain (NOD), leucine-rich repeat (LRR), pyrin domain-containing protein 3 (NLRP3), NLR family caspase recruitment domain (CARD)-containing protein 4 (NLRC4), absent in melanoma 2 (AIM2), and cytokine precursors. And inflammasome requires the second phase triggered by pathogen- and danger-associated molecular patterns, an activation step, to assemble a cytosolic sensor, an adaptor [apoptosis-associated speck-like protein containing a CARD (APC)], and an effector (pro-caspase-1) into inflammasome complex, and to secrete IL-1β and IL-18 (Kanneganti, 2015; Rathinam and Fitzgerald, 2016; Swanson et al., 2019; Yang et al., 2019).

Interestingly, it was found that MB inhibits inflammasome activation by interrupting both steps. Ahn et al. elucidated the relation between MB and inflammasome for the first time, demonstrating that MB suppresses both canonical and non-canonical processes by decreasing dose-dependently the expression of NLRP3, pro-IL-1β and caspase-1, as well as reactive oxygen species (ROS) production (Ahn et al., 2017).

Similarly, neuroinflammation triggered by spinal cord injury (SCI) was alleviated by the MB-mediated inhibition of NLRP3 and NLRC4 inflammasome formation in microglia. In SCI animals, following MB administration, their locomotive activities were ameliorated and inflammatory cytokine levels (IL-1β, IL-6, and TNF-α) were also diminished (Lin et al., 2017). Overall MB-mediated anti-inflammation pathways are summarized in Table 1.

**TABLE 1:** MB-mediated multiple anti-inflammation pathways.

Pathways	Changes in key elements	Final results	References
MB-NOS/NO	iNOS expression ↓ NO production ↓ TGF-β receptor expression ↑	cartilage matrix and proteoglycan degradation ↓ (in cultured human cartilage explants)	Cohen et al., 2000
	iNOS expression ↓ NO production ↓ IL-1β, IL-6, TNF-α expression ↓	tissue injury and edema ↓ (in rats with acetic acid-induced colitis)	Dinc et al., 2015
	iNOS expression ↓ NO production ↓	NF-κB binding to iNOS promoter ↓ (in mouse organs and cultured cells)	Huang et al., 2015
	nNOS expression ↓ MMP-2, MMP-3 expression ↓ PEDF expression ↑	inner retinal thickness ↓ gliosis ↓ retinal angiogenesis ↓ (in retinopathy rats)	Rey-Funes et al., 2016
MB-P2 × 3	P2 × 3R expression ↓ IL-1β, IL-6, IL-8, TNF-α expression ↓	swelling ratio in the inflamed knee ↓ weight distribution in hind paws ↑ (in OA rabbits)	Li et al., 2018
MB-lncRNA	CILinc02 expression ↓ IL-1, IL-6, and IL-17 expression ↓	chondrocyte degradation ↓ inflammation ↓ (in human cartilage tissues and primary cells)	Zheng and Li, 2019
MB-inflammasome	NLRP3, pro-IL-1β expression ↓ IL-1β, IL-18, caspase-1 expression ↓ mitochondrial ROS production ↓	inflammasome activation ↓ (in bone marrow-derived macrophages)	Ahn et al., 2017
	NLRP3 expression ↓ ROS production ↓ IL-1β, IL-6, IL-18, TNF-α expression ↓	NLRP3 and NLRC4 inflammasome formation ↓ neuroinflammation ↓ locomotive function ↑ (in cultured microglia and spinal cord injured rats)	Lin et al., 2017

## MB and Sodium Current Reduction

### Early Studies for MB and *I*_*NA*_

**FIGURE 2:**
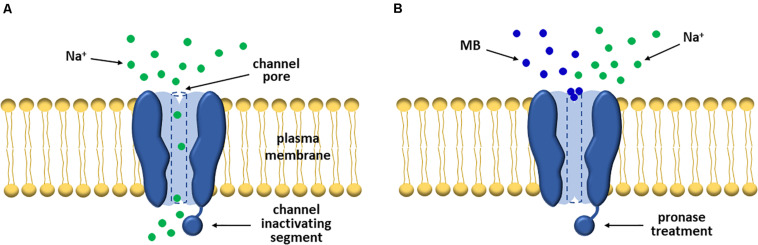
MB significantly attenuates sodium currents by blocking VGSCs. **(A)** In general, VGSCs allow sodium ions to flow into the cell in the activated state. **(B)** However, early researchers found that the gate and sodium currents of the channels were markedly suppressed post-MB treatment. And notably, this event was maintained even after pronase treatment. Thus, they interpreted this event as MB functions as a pore blocker rather than an inactivation enhancer.

It is an old story that MB is involved in the reduction of excitatory synaptic currents. In earlier periods, Armstrong, Croop, and Starkus conducted pioneering experiments to explore the inactivation of sodium channels and their electrophysiological profiles using an artificial inactivation model established by administering MB into squid giant axons. The dyes blocked efficiently both the *I*_*g*_ (gate current) and *I*_*NA*_, as well as simulated the channel inactivation even after pronase, an agent of eliminating normal inactivation, treatment (Armstrong and Croop, 1982). Similarly, a subsequent study showed that MB weakened sodium currents in both normal and pronase-treated crayfish giant axons (Starkus et al., 1984). In the two studies, MB was believed to be a gate-immobilizing open-pore blocker rather than an inactivation enhancer, since directly blocking sodium pores rather than promoting or speeding the inactivation process (Figure 2; Armstrong and Croop, 1982; Starkus et al., 1984).

### Altered VGSC-Dependent Currents and Neural Firing Rates

Zhang et al. (2010) explored the effect of MB on sodium currents and found that MB alters VGSC-mediated electrophysiological events. MB application diminished the peak amplitude of *I*_*NA*_ up to 45% at 100 μM, number of repetitive firings, and V_*max*_ (AP upstroke velocity) in rat hippocampal slices. In addition, it accelerated *I*_*NA*_ inactivation and even retarded the shift from inactivation to recovery state.

Importantly, Lee et al. (2021) performed the first *in vivo* experiments to investigate the link between MB-mediated neural firing patterns and pain reduction in rats using *in vivo* single nerve recordings and behavioral studies. This study showed dramatic results that neural firing rates significantly decreased in a dose-dependent manner after MB administration and, notably, this event lasted longer than that of lidocaine, showing anesthetic-like firing patterns. Ultimately, MB improved pain behaviors in rodents. These results demonstrate that MB abates pain by markedly suppressing neural excitability (Lee et al., 2021).

#### Silenced Excitable Neurons as a Critical Cause of Pain Relief

Exciting neurons is the most fundamental step to convey signals and to relay or cause several subsequent responses. Moreover, VGSCs are directly engaged in AP generation and affect the resultant reactions including signal propagation, opening other channels, vesicle release, cell-to-cell communication, and other related responses (Kruger and Isom, 2016; Wang et al., 2017). Accordingly, it is natural to postulate that VGSCs and AP generation play a crucial role even in pain transmission (Dubin and Patapoutian, 2010; Bennett et al., 2019). Interestingly, multiple evidence demonstrates that MB silences excitable cells (or nerves) by significantly attenuating *I*_*NA*_ and AP production, which ultimately leads to pain relief (Armstrong and Croop, 1982; Starkus et al., 1984, 1993; Zhang et al., 2010; Lee et al., 2021). Therefore, these two crucial electrophysiological activities, weakened sodium currents and decreased neural firing rates, are possible causes that can elucidate how MB contributes to pain relief.

## MB and Denervation

### Early Cases for MB Application

Denervation is defined as loss or interruption of nerve connection to an organ, which thereby may contribute to pain relief by making nerves shut off sensory transmission. In history, MB began to rear its head for denervation firstly to treat itch. In 1968, Professor Rygick in the Moscow University reported for the first time an effect of MB on chronic pruritus ani (PA), characterized by severe itching in the perianal area, noting that patients with chronic PA was significantly improved after subcutaneous MB administration (Rygick, 1968). In 1979, Wollock and Dintsman inspired by Rygick’s work conducted also an intervention to treat intractable PA locally administering MB into the perianal skin and produced remarkable results that eight out of nine patients were completely improved post-MB treatment. Importantly, Eusebio et al. conducted an electron microscopic investigation aimed at the perianal skin of intractable PA patients treated with subcutaneous MB and no distinct nerve endings were detected in the samples even after a long-term (7 years) follow-up. Thus, they interpreted that this improvement was attributed to death of nerve endings connected to the perianal skin, that is, denervation (Eusebio et al., 1990). Most recently, PA patients treated with MB showed satisfactory scores at 6-week, as well as 3-year, follow-ups. The recurrence rate was low (7.5%) even three years after MB treatment. In the study, MB was also considered as a critical agent to sever nerve endings (Kim et al., 2019).

### Intradiscal MB Injection and Debates

#### The Novel Intervention to Treat Chronic Pain

Similar to the itching cases, MB was applied to inflamed intervertebral disks (IVDs) in CDLBP patients. In 2007, Peng et al. (2007) reported encouraging outcomes in the treatment of CDLBP using a minimal invasive method, intradiscal MB injection. They believed that MB has a capacity to mitigate pain in the patients by blocking nerve conduction or destroying nerve endings around the painful disks that may be vascularized and extensively innervated owing to disk degeneration. This was a first prospective clinical trial conducted for examining the pain-relieving effect of MB in human subjects. In this study, MB was administered into the affected disks of CDLBP patients using a discographic needle (1 ml of 1% MB), then pain intensity and disability of the patients were measured by visual analog scale (VAS) and oswestry disability index (ODI). As a result, most patients were evidently or completely improved and there were no side effects or complications during long-term follow-up periods (12-23 months) (Peng et al., 2007). After four years, Peng et al. (2010) conducted a randomized placebo-controlled trial in 72 eligible participants, who accepted intradiscal MB injection or placebo treatment. For numerical rating scale (NRS) and ODI scores, there were dramatic or obvious improvements in most patients treated with MB. Of note, these events lasted even to the subsequent follow-up visits at 12 and 25 months.

#### Controversies and Refutations for Intradiscal MB Application

However, their outcomes were immediately challenged and debated over a more fundamental issue. Bogduk (2010) explained that cultural factors may affect the manner in which patients report their outcomes, and Chinese patients may report their physical conditions more favorably to physicians or assessors than United States or British patients do. Another commentary was about the safety of MB. MB has a potent neurotoxic effect and can be leaked to the spinal canal through annular tears during intradiscal administration since discogenic pain is associated with annular tears (Levine and Richeimer, 2011). Lastly, a researcher argued that there is no LBP treatment that can immunize against new LBP episodes, emphasizing that discography accelerates disk degeneration after years (Schiltenwolf et al., 2011).

In 2019, an article directly refuted the previous results of Peng et al. (2010) designing a study protocol almost identical to their previous study to verify their outcomes (Kallewaard et al., 2019). As a result, there were no significant differences between MB plus lidocaine treatment group and placebo plus lidocaine group after NRS, ODI, quality of life (QOL), and VAS measurement. They concluded that the outcomes of the previous study were not able to be reproduced (Kallewaard et al., 2019).

#### Short-Term Effect of Intradiscal MB Application on Chronic Low Back Pain

Over years after Peng et al. (2010), there have been multiple attempts to perform intradiscal MB injection in CDLBP patients. Kim et al. revealed that following intradiscal MB injection, there was a significant decrease in VAS (1, 3, and 6 months) and ODI (one and three months) scores in 20 CDLBP patients, but, one year after intervention, such outcomes were reproduced only in five patients (Kim et al., 2012). Accordingly, the patients showed short-term improvement (three or six months) after intervention. Gupta et al. showed a relatively low success rate (13%) in eight CDLBP patients after a single intradiscal MB injection (Gupta et al., 2012). Levi et al. revealed that there were very limited benefits in the VAS and ODI scores in 16 CDLBP patients. Only a few patients (25% or less) met the criteria for success at the follow-up periods (Levi et al., 2014). Lastly, Zhang X. et al. (2016) reported the short-term clinical outcomes in CDLBP patients. Their NRS and ODI scores were significantly decreased at 1- to 6-month follow-ups. Imaging experiments showed that the mean apparent diffusion coefficient (ADC) and T2 values significantly increased at 6- and 12-month, but not 3-month, follow-ups.

Collectively, it was found that MB is effective in CDLBP patients at least in a short period (until 3 or 6 months after treatment), which is also supported by the most recent review report (Deng et al., 2021). Similarly, radiofrequency (RF) denervation also resulted in short-term improvement (4 weeks or 6 months) in patients with chronic LBP (Leclaire et al., 2001; Nath et al., 2008; Al-Najjim et al., 2018). In addition, epidermal nerve fibers were regenerated in healthy humans about 100 days after capsaicin-induced denervation (Polydefkis et al., 2004). Regarding knee osteoarthritis, RF denervation contributed to pain reduction in patients with inflamed knee joints and their joint function was ameliorated. This improvement lasted 6 and 3 months, respectively (Wang R. et al., 2019). Based on these results, it is believed that denervating effects do not exceed 6 months. Accordingly, the short-term improvement observed in CDLBP patients is interpreted reasonable. Overall results of the studies were summarized in Table 2.

**TABLE 2:** Overall results of intradiscal MB Injection in CDLBP Patients.

Case report	Number of patients	Measurements	Duration of pain relief	Success rate
Peng et al., 2007	24	VAS, ODI	23 mo	87% at 3, 6, and 12 (or more) mo
Peng et al., 2010	36 (MB) 36 (placebo)	NRS, ODI	24 mo	89% at 6, 12, and 24 mo
Kim et al., 2012	20	VAS, ODI	12 mo	55% and 20% at 3 and 12 mo
Gupta et al., 2012	8	retrospective analysis	12 mo	13% at 12 mo
Levi et al., 2014	16	VAS, ODI	6 mo	25%, 21%, and 25% at 1, 2, and 6 mo, respectively
Zhang X. et al., 2016	33	NRS, ODI	12 mo	81, 75, 63 and 54% at 1, 3, 6, and 12 mo, respectively
Kallewaard et al., 2019	40 (MB) 41 (placebo)	NRS, PGIC, VAS, ODI, QoL	6 mo	15(12)%, 25(20)%, and 35(25)% at 6 wk, 3 mo, and 6 mo in NRS (PGIC), respectively

*PGIC, patent global impression of change; wk, week(s); mo, month(s).*

## MB and Toxicity Issues

### Serotonin Toxicity

#### The Contribution of MB to Serotonin Toxicity as a Potent MAO Inhibitor

In the last 20 years, it has been reported that depressive or anxiety patients taking serotonergic medications experienced ST, also referred to as serotonin syndrome, after MB administration. The investigators and medical doctors have believed that the toxicity is deeply linked with the synergism of MB and the medications, and that such patients may be more vulnerable to ST (Ng et al., 2008; Ng and Cameron, 2010; Kapadia et al., 2016; Wolvetang et al., 2016).

ST is characterized by the excessive accumulation of serotonin into the body, which, ultimately, leads to neuromuscular hyperexcitability by the excessive serotonergic agonism of the central and peripheral nervous system, whose clinical findings include agitation, tremor, inducible and ocular clonus, diaphoresis, hyperreflexia, hypertonia, and hyperthermia (over 38°C) (Boyer and Shannon, 2005). A great number of case reports showed that these events occurred predominantly in mental patients who had been taking serotonergic antidepressant medications including fluoxetine [a selective serotonin reuptake inhibitor (SSRI)] (Martindale and Stedeford, 2003; Kapadia et al., 2016), paroxetine, a SSRI (Bach et al., 2004; Mihai et al., 2007; Ng et al., 2008; Shanmugam et al., 2008; Schwiebert et al., 2009; Wolvetang et al., 2016), venlafaxine [a selective serotonin and norepinephrine reuptake inhibitor (SSNRI)] (Majithia and Stearns, 2006), citalopram, a SSRI (Mathew et al., 2006; Pollack et al., 2009), duloxetine, a SSNRI (Rowley et al., 2009), and clomipramine [a serotonin reuptake inhibitor (SRI)] (Khan et al., 2007), and have been commonly interpreted to be attributed to the reaction of MB as a potent monoamine oxidase A (MAO-A) inhibitor (Gillman, 2011; Top et al., 2014). In effect, MAO has a part in the degradation process of a diversity of monoamines such as serotonin, epinephrine, norepinephrine, dopamine, and histamine (Yeung et al., 2019; Floris et al., 2020). In this regard, MB augments serotonin levels in the synaptic cleft by suppressing the activity of MAO, which, furthermore, may lead to ST along with the use of SRIs, SSRIs, and SSNRIs due to over-enhanced serotonergic transmission (Zuschlag et al., 2018).

#### The Risk of Intravenous MB Application in Patients Taking Serotonergic Medications

Previous studies showed that ST was precipitated after intravenous MB administration predominantly at doses of 5-7.5 mg/kg except for the case of a 65-year-old woman (1.75 mg/kg) (Mihai et al., 2007; Ng et al., 2008). More importantly, Gillman reported that even a low dose of MB (0.75 mg/kg) administered via the intravenous route may reach peak plasma concentration and cause CNS toxicity (or ST) in those being treated with the medications facilitating serotonergic transmission (Schwiebert et al., 2009; Gillman, 2011). The drug safety update, a newsletter issued by the Medicines and Healthcare products Regulatory Agency (MHRA), also informed that patients who have recently treated with serotonergic antidepressants should avoid intravenous MB and be closely observed if administered with MB, and that intravenous MB can be approved only for patients with drug-induced methemoglobinemia at a dose of 1–2 mg/kg (MHRA, 2009). Oral administration of MB is not likely to cause ST since the MB concentration in blood and brain was significantly higher after intravenous, compared to oral, administration (Peter et al., 2000; Top et al., 2014). Therefore, we need to avoid intravenous route and consider about proper dosage to prevent this toxicity event.

### Other Issues

A previous study demonstrated that MB deteriorated the dendritic arbor of isolated neurons and was engaged in cell death in a dose-dependent manner (no neurotoxic effects at concentrations of 0.25 μM or less) (Vutskits et al., 2008). More recently, it was found that MB has a detrimental effect on the viability of nucleus pulposus and annulus fibrosus cells. But MB at lower doses had little effect on both (Wang X. et al., 2019; Zhang et al., 2019). Thus, these results imply that high-dose MB may impair them, thus we need to consider about proper dosage in the clinical setting.

## Discussion

**FIGURE 3:**
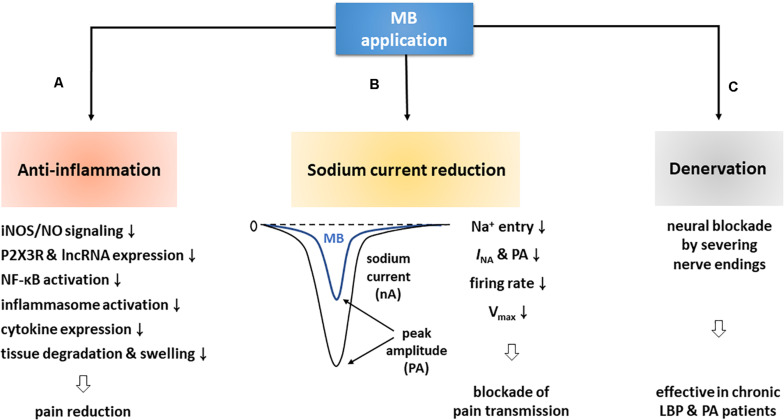
MB contributes to pain reduction via three major routes. **(A)** First of all, MB is deeply involved in anti-inflammation. MB application blocks iNOS/NO signaling by downregulating iNOS, and suppresses P2 × 3R and lncRNA expression, NF-κB activation, and inflammasome formation, which thereby decreases inflammatory cytokine levels. These events are ultimately followed by pain reduction with the prevention of tissue degradation and swelling. **(B)** In addition, MB application attenuates neuronal excitability by decreasing *I*_*NA*_ and firing rates. These altered electrophysiological properties may contribute to pain relief by blocking synaptic transmission. **(C)** Lastly, MB application improved chronic PA and LBP. An electron microscopic experiment demonstrated that such efficacy was due to the death of nerve endings.

In the present review, we illustrated MB-driven analgesic effects and their possible mechanisms based on key clinical and experimental studies. In effect, MB is much closely involved in pain relief via the following major actions: anti-inflammation, sodium current reduction, and denervation (Figure 3).

First of all, MB is deeply related to anti-inflammation. Notably, it triggers a variety of anti-inflammatory pathways and functions to decrease the expression level of pro-inflammatory cytokines in a variety of ways, including inhibition of iNOS/NO signaling, downregulation of P2XR and lncRNA, and interruption of priming and activation steps required for inflammasome complex formation and activation. Ultimately, these anti-inflammatory responses contribute to restoring inflamed tissues and lessening pain (Table 1; Vutskits et al., 2008; Dinc et al., 2015; Ahn et al., 2017; Li et al., 2018; Zheng and Li, 2019). In addition, MB reduces the amplitude of *I*_*NA*_, accelerates the *I*_*NA*_ inactivation, delays the shift from inactivation to recovery state in neurons, and importantly decreases neural firing rates, which can therefore alleviate pain by impeding VGSC-mediated neuronal excitability (Zhang et al., 2010; Lee et al., 2021). Lastly, MB is involved in denervation. MB has been believed to have a neurolytic effect and thus to destroy dermal nerve endings for years. Of note, this effect was confirmed by an electron microscopic experiment (Eusebio et al., 1990; Etter and Myers, 2002). In clinical cases, CDLBP patients were improved for at least 3 or 6 months after intradiscal MB injection and RF denervation (Kim et al., 2012; Maas et al., 2015; Zhang X. et al., 2016; Al-Najjim et al., 2018).

However, there are toxicity problems with MB application. Patients being treated with serotonergic medications are likely to suffer ST after intravenous MB administration (mainly at doses of 5-7.5 mg/kg) due to the synergistic effect of MB and the medications. In addition, MB is able to damage cultured neurons (Vutskits et al., 2008) and NP and AF cells in a dose-dependent manner (Wang X. et al., 2019; Zhang et al., 2019). Therefore, we should find proper dose levels and administration routes to protect MB-induced adverse events.

MB is a blue dye that had been used at first for textile manufacturing. Similarly, at the present day, it is applied as a dye to mark and visualize a certain tissue or region in the clinical and experimental settings. However, astonishingly, MB has been also used in biological and medical studies for a long time. Importantly, this review highlighted the relation between MB and pain reduction and made an effort to inform the relevant mechanisms. MB is engaged in pain reduction via diverse routes, but there is a still lack of scientific evidence. There are still a lot of blanks to be filled in the anti-inflammatory pathways and remain possible links to be uncovered between MB and pain-related receptors.

Modern medicine lies in the positivist tradition, which implies that medicine must be based on a solid foundation and the foundation can be constructed by the faithful observation of life phenomena (Cabanis, 1803). In addition, the certainty of medical facts can be guaranteed by that of experimental science such as experimental certainty and logical certainty. The former can be obtained by senses of observant subjects, the latter by intellectual action of humans (Bouillaud, 1836). With regard to the relation between MB and pain control, a solid foundation has not been established yet, although MB intercalates into critical signal pathways to reduce inflammation and pain. Thus, we need still further investigations to build the foundation and reach the certainty of medical facts.

Lessening pain is one of the salient issues in the medical profession, which may also determine our QoL. A great number of medications have been explored and are now applied to patients to relieve pain. Given the actions of MB in pathological or inflammatory milieus, this review offers a strong possibility that this dye may be developed as a new therapeutic agent for pain patients.

## Author Contributions

First of all, HH and SL set up the concept of this study, were involved in its overall design, and wrote this manuscript. Moreover, SL put a lot of effort into acquiring and analyzing the relevant articles and data, and made tables and figures to illustrate more clearly methylene blue-mediated events and mechanisms. Both authors contributed to completing this manuscript.

## Conflict of Interest

The authors declare that the research was conducted in the absence of any commercial or financial relationships that could be construed as a potential conflict of interest.

## References

[B1] Ahn H. Kang S. G. Yoon S. I. Ko H. J. Kim P. H. Hong E. J. (2017). Methylene blue inhibits NLRP3, NLRC4, AIM2, and non-canonical inflammasome activation. *Sci. Rep.* 7:12409. 10.1038/s41598-017-12635-6 PMC5622101 28963531

[B2] Al-Najjim M. Shah R. Rahuma M. Gabbar O. A. (2018). Lumbar facet joint injection in treating low back pain: radiofrequency denervation versus SHAM procedure. Systematic review. *J. Orthop.* 15 1–8. 10.1016/j.jor.2017.10.001 29167604 PMC5686472

[B3] Armstrong C. M. Croop R. S. (1982). Simulation of Na channel inactivation by thiazine dyes. *J Gen Physiol* 80 641–662. 10.1085/jgp.80.5.641 6294219 PMC2228641

[B4] Aubrey B. J. Kelly G. L. Janic A. Herold M. J. Strasser A. (2018). How does p53 induce apoptosis and how does this relate to p53-mediated tumour suppression? *Cell Death Differ.* 25 104–113. 10.1038/cdd.2017.169 29149101 PMC5729529

[B5] Bach K. K. Lindsay F. W. Berg L. S. Howard R. S. (2004). Prolonged postoperative disorientation after methylene blue infusion during parathyroidectomy. *Anesth Analg* 99 1573–1574. 10.1213/01.ane.0000134860.73875.cf 15502068

[B6] Bennett D. L. Clark A. J. Huang J. Waxman S. G. Dib-Hajj S. D. (2019). The role of voltage-gated sodium channels in pain signaling. *Physiol. Rev*, 99 1079–1151. 10.1152/physrev.00052.2017 30672368

[B7] Bernier L. P. Ase A. R. Seguela P. (2018). P2X receptor channels in chronic pain pathways. *Br. J. Pharmacol.* 175 2219–2230. 10.1111/bph.13957 28728214 PMC5980614

[B8] Bogduk N. (2010). A cure for back pain? *Pain* 149 7–8. 10.1016/j.pain.2009.05.022 20129734

[B9] Bouillaud J. (1836). *Essai sur la Philosophie Médicale et sur les Généralités de la Clinique Médicale.* Oxford: Université d’Oxford. De Just Rouvier et E. Le Bouvier.

[B10] Boyer E. W. Shannon M. (2005). The serotonin syndrome. *N. Engl. J. Med.* 352 1112–1120. 15784664 10.1056/NEJMra041867

[B11] Cabanis P. J. G. (1803). *Du Degré de Certitude de la Médecine.* Paris: Crapelet.

[B12] Chen J. Ao L. Yang J. (2019). Long non-coding RNAs in diseases related to inflammation and immunity. *Ann. Transl. Med.* 7:494. 10.21037/atm.2019.08.37 31700930 PMC6803193

[B13] Chen W. Walwyn W. Ennes H. S. Kim H. Mcroberts J. A. Marvizón J. C. (2014). BDNF released during neuropathic pain potentiates NMDA receptors in primary afferent terminals. *Eur. J. Neurosci.* 39 1439–1454. 10.1111/ejn.12516 24611998 PMC4122572

[B14] Chu Y.-C. Guan Y. Skinner J. Raja S. N. Johns R. A. Tao Y.-X. (2005). Effect of genetic knockout or pharmacologic inhibition of neuronal nitric oxide synthase on complete Freund’s adjuvant-induced persistent pain. *Pain* 119 113–123. 10.1016/j.pain.2005.09.024 16297560

[B15] Cohen N. Robinson D. Ben-Ezzer J. Hemo Y. Hasharoni A. Wolmann Y. (2000). Reduced NO accumulation in arthrotic cartilage by exposure to methylene blue. *Acta Orthop. Scand.* 71 630–636. 10.1080/000164700317362299 11145393

[B16] Demir I. E. Heinrich T. Carty D. G. Saricaoglu ØC. Klauss S. Teller S. (2019). Targeting nNOS ameliorates the severe neuropathic pain due to chronic pancreatitis. *EBioMedicine* 46 431–443. 10.1016/j.ebiom.2019.07.055 31401195 PMC6711864

[B17] Deng M. Huang H. Ma Y. G. Zhou Y. Chen Q. Xie P. (2021). Intradiskal injection of methylene blue for discogenic back pain: a meta-analysis of randomized controlled trials. *J. Neurol. Surg. A Cent Eur. Neurosurg.* 82 161–165. 10.1055/s-0040-1721015 33477188

[B18] Deng S. Yu K. Zhang B. Yao Y. Wang Z. Zhang J. (2015). Toll-like receptor 4 promotes NO synthesis by upregulating GCHI expression under oxidative stress conditions in sheep Monocytes/Macrophages. *Oxid. Med. Cell.Longev.* 2015:359315. 10.1155/2015/359315 PMC4630417 26576220

[B19] Dinc S. Caydere M. Akgul G. Yenidogan E. Hucumenoglu S. Rajesh M. (2015). Methylene Blue inhibits the inflammatory process of the acetic acid-induced colitis in the rat colonic mucosa. *Int. Surg.* 100 1364–1374. 10.9738/intsurg-d-15-00118.1 26062761

[B20] Dubin A. E. Patapoutian A. (2010). Nociceptors: the sensors of the pain pathway. *J. Clin. Invest.* 120 3760–3772. 10.1172/jci42843 21041958 PMC2964977

[B21] Etter L. Myers S. A. (2002). Pruritus in systemic disease: mechanisms and management. *Dermatol. Clin.* 20 459–472. 10.1016/s0733-8635(02)00011-6 12170879

[B22] Eusebio E. B. Graham J. Mody N. (1990). Treatment of intractable pruritus ani. *Dis Colon Rectum.* 33 770–772. 10.1007/bf02052324 2390913

[B23] Floris G. Cadeddu R. Bortolato M. (2020). “The effects of serotonin degradation on psychopathology: role of monoamine oxidase,” in *Handbook of the Behavioral Neurobiology of Serotonin*, eds Müller C. P. Cunningham K. A. (Cambridge, MA: Academic Press), 267–278. 10.1016/b978-0-444-64125-0.00014-1

[B24] Funakoshi A. Tatsuno K. Shimauchi T. Fujiyama T. Ito T. Tokura Y. (2019). Cholecystokinin downregulates psoriatic inflammation by its possible self-regulatory effect on epidermal keratinocytes. *J. Immunol.* 202:2609. 10.4049/jimmunol.1801426 30902899

[B25] Gillman P. K. (2011). CNS toxicity involving methylene blue: the exemplar for understanding and predicting drug interactions that precipitate serotonin toxicity. *J. Psychopharmacol.* 25 429–436. 10.1177/0269881109359098 20142303

[B26] Gupta G. Radhakrishna M. Chankowsky J. Asenjo J. F. (2012). Methylene blue in the treatment of discogenic low back pain. *Pain Physician* 15 333–338. 10.36076/ppj.2012/15/333 22828687

[B27] Harth M. Nielson W. R. (2019). Pain and affective distress in arthritis: relationship to immunity and inflammation. *Expert Rev. Clin. Immunol.* 15 541–552. 10.1080/1744666x.2019.1573675 30669892

[B28] Huang C. Tong L. Lu X. Wang J. Yao W. Jiang B. (2015). Methylene blue attenuates iNOS induction through suppression of transcriptional factor binding amid iNOS mRNA transcription. *J. Cell Biochem.* 116 1730–1740. 10.1002/jcb.25132 25736558

[B29] Jiang M. Wang H. Liu Z. Lin L. Wang L. Xie M. (2020). Endoplasmic reticulum stress-dependent activation of iNOS/NO-NF-κB signaling and NLRP3 inflammasome contributes to endothelial inflammation and apoptosis associated with microgravity. *FASEB J.* 34 10835–10849. 10.1096/fj.202000734r 32592441

[B30] Kallewaard J. W. Wintraecken V. M. Geurts J. W. Willems P. C. Van Santbrink H. Terwiel C. T. M. (2019). A multicenter randomized controlled trial on the efficacy of intradiscal methylene blue injection for chronic discogenic low back pain: the IMBI study. *Pain* 160 945–953. 10.1097/j.pain.0000000000001475 30730862

[B31] Kanneganti T. D. (2015). The inflammasome: firing up innate immunity. *Immunol. Rev.* 265 1–5. 10.1111/imr.12297 25879279 PMC4701203

[B32] Kapadia K. Cheung F. Lee W. Thalappillil R. Florence F. B. Kim J. (2016). Methylene blue causing serotonin syndrome following cystocele repair. *Urol. Case Rep.* 9 15–17. 10.1016/j.eucr.2016.07.012 27617215 PMC5011181

[B33] Kaski S. W. White A. N. Gross J. D. Siderovski D. P. (2021). Potential for kappa-opioid receptor agonists to engineer nonaddictive analgesics: a narrative review. *Anesth. Anal.* 132 406–419. 10.1213/ane.0000000000005309 33332902 PMC7992303

[B34] Keppel Hesselink J. M. (2020). Rediscovery of ceruletide, a CCK agonist, as an analgesic drug. *J. Pain Res.* 13 123–130. 10.2147/jpr.s232714 32021401 PMC6970274

[B35] Khan M. A. North A. P. Chadwick D. R. (2007). Prolonged postoperative altered mental status after methylene blue infusion during parathyroidectomy: a case report and review of the literature. *Ann. R. Coll. Surg. Engl.* 89 W9–W11. 10.1308/147870807X160434 PMC1964586 17346391

[B36] Kim J. H. Kim D. H. Lee Y. P. (2019). Long-term follow-up of intradermal injection of methylene blue for intractable, idiopathic pruritus ani. *Tech. Coloproctol.* 23 143–149. 10.1007/s10151-019-01934-x 30734161 PMC6440940

[B37] Kim K. H. Kim J. I. Han J. A. Choe M. A. Ahn J. H. (2011). Upregulation of neuronal nitric oxide synthase in the periphery promotes pain hypersensitivity after peripheral nerve injury. *Neuroscience* 190 367–378. 10.1016/j.neuroscience.2011.05.064 21664432

[B38] Kim S. H. Ahn S. H. Cho Y. W. Lee D. G. (2012). Effect of intradiscal methylene blue injection for the chronic discogenic low back pain: one year prospective follow-up study. *Ann. Rehabil. Med.* 36 657–664. 10.5535/arm.2012.36.5.657 23185730 PMC3503941

[B39] Kõks S. Fernandes C. Kurrikoff K. Vasar E. Schalkwyk L. C. (2008). Gene expression profiling reveals upregulation of Tlr4 receptors in Cckb receptor deficient mice. *Behav. Brain Res.* 188 62–70. 10.1016/j.bbr.2007.10.020 18054398

[B40] Kourosh-Arami M. Hosseini N. Mohsenzadegan M. Komaki A. Joghataei M. T. (2020). Neurophysiologic implications of neuronal nitric oxide synthase. *Rev. Neurosci.* 31 617–636. 10.1515/revneuro-2019-0111 32739909

[B41] Kruger L. C. Isom L. L. (2016). Voltage-Gated Na+ Channels: not just for conduction. *Cold Spring Harbor Perspect. Biol.* 8:a029264. 10.1101/cshperspect.a029264 27252364 PMC4888818

[B42] Lacagnina M. J. Watkins L. R. Grace P. M. (2018). Toll-like receptors and their role in persistent pain. *Pharmacol. Ther.* 184 145–158. 10.1016/j.pharmthera.2017.10.006 28987324 PMC5858962

[B43] Leclaire R. Fortin L. Lambert R. Bergeron Y. M. Rossignol M. (2001). Radiofrequency facet joint denervation in the treatment of low back pain: a placebo-controlled clinical trial to assess efficacy. *Spine* 26 1411–1416. discussion 1417, 10.1097/00007632-200107010-00003 11458140

[B44] Lee S. W. Moon S. W. Park J. S. Suh H. R. Han H. C. (2021). Methylene blue induces an analgesic effect by significantly decreasing neural firing rates and improves pain behaviors in rats. *Biochem. Biophys. Res. Commun.* 541 36–42. 10.1016/j.bbrc.2021.01.008 33465740

[B45] Leiper J. Nandi M. (2011). The therapeutic potential of targeting endogenous inhibitors of nitric oxide synthesis. *Nat. Rev. Drug Discov.* 10 277–291. 10.1038/nrd3358 21455237

[B46] Levi D. S. Horn S. Walko E. (2014). Intradiskal methylene blue treatment for diskogenic low back pain. *PM R* 6 1030–1037. 10.1016/j.pmrj.2014.04.008 24780850

[B47] Levine R. Richeimer S. H. (2011). Spinal methylene blue is hazardous. *PAIN* 152 952–953. 10.1016/j.pain.2011.01.009 21282009

[B48] Lewin M. R. Walters E. T. (1999). Cyclic GMP pathway is critical for inducing long–term sensitization of nociceptive sensory neurons. *Nat. Neurosci.* 2 18–23. 10.1038/4520 10195175

[B49] Li G. Jiang H. Zheng C. Zhu G. Xu Y. Sheng X. (2017). Long noncoding RNA MRAK009713 is a novel regulator of neuropathic pain in rats. *PAIN* 158 2042–2052. 10.1097/j.pain.0000000000001013 28708759

[B50] Li H. Liu S. Wang Z. Zhang Y. Wang K. (2020). Hydrogen sulfide attenuates diabetic neuropathic pain through NO/cGMP/PKG pathway and μ-opioid receptor. *Exp. Biol. Med.* 245 823–834. 10.1177/1535370220918193 32268802 PMC7273888

[B51] Li X. Tang C. Wang J. Guo P. Wang C. Wang Y. (2018). Methylene blue relieves the development of osteoarthritis by upregulating lncRNA MEG3. *Exp. Ther. Med.* 15 3856–3864. 29581742 10.3892/etm.2018.5918 PMC5863598

[B52] Lin Z. H. Wang S. Y. Chen L. L. Zhuang J. Y. Ke Q. F. Xiao D. R. (2017). Methylene blue mitigates acute neuroinflammation after spinal cord injury through inhibiting nlrp3 inflammasome activation in microglia. *Front. Cell Neurosci.* 11:391. 10.3389/fncel.2017.00391 29311826 PMC5732444

[B53] Lundberg J. O. Weitzberg E. Gladwin M. T. (2008). The nitrate-nitrite-nitric oxide pathway in physiology and therapeutics. *Nat. Rev. Drug Discov.* 7 156–167. 10.1038/nrd2466 18167491

[B54] Luo Z. D. Cizkova D. (2000). The role of nitric oxide in nociception. *Curr. Rev. Pain* 4 459–466. 10.1007/s11916-000-0070-y 11060592

[B55] Maas E. T. Ostelo R. W. Niemisto L. Jousimaa J. Hurri H. Malmivaara A. (2015). Radiofrequency denervation for chronic low back pain. *Cochrane Database Syst. Rev.* CD008572. 10.1002/14651858.CD008572.pub2 PMC8782593 26495910

[B56] Machelska H. Labuz D. Przewłocki R. Przewłocka B. (1997). Inhibition of nitric oxide synthase enhances antinociception mediated by mu, delta and kappa opioid receptors in acute and prolonged pain in the rat spinal cord. *J. Pharmacol. Exp. Ther.* 282 977–984. 9262366

[B57] Majithia A. Stearns M. P. (2006). Methylene blue toxicity following infusion to localize parathyroid adenoma. *J. Laryngol. Otol.* 120 138–140. 10.1017/s0022215105005098 16359577

[B58] Martindale S. J. Stedeford J. C. (2003). Neurological sequelae following methylene blue injection for parathyroidectomy. *Anaesthesia* 58 1041–1042. 10.1046/j.1365-2044.2003.03415_23.x 12969068

[B59] Mathew S. Linhartova L. Raghuraman G. (2006). Hyperpyrexia and prolonged postoperative disorientation following methylene blue infusion during parathyroidectomy. *Anaesthesia* 61 580–583. 10.1111/j.1365-2044.2006.04619.x 16704594

[B60] Matsuda M. Huh Y. Ji R. R. (2019). Roles of inflammation, neurogenic inflammation, and neuroinflammation in pain. *J. Anesth.* 33 131–139. 10.1007/s00540-018-2579-4 30448975 PMC6813778

[B61] MHRA, (2009). *Methylthioninium Chloride (methylene blue): Update on Central Nervous System (CNS) Toxicity.* London: Medicines and Healthcare products Regulatory Agency, 2.

[B62] Miclescu A. A. Svahn M. Gordh T. E. (2015). Evaluation of the protein biomarkers and the analgesic response to systemic methylene blue in patients with refractory neuropathic pain: a double-blind, controlled study. *J. Pain Res.* 8 387–397. 10.2147/jpr.s84685 26213475 PMC4509536

[B63] Mihai R. Mitchell E. W. Warwick J. (2007). Dose-response and postoperative confusion following methylene blue infusion during parathyroidectomy. *Can. J. Anaesth.* 54 79–81. 10.1007/bf03021907 17197475

[B64] Mukherjee P. Cinelli M. A. Kang S. Silverman R. B. (2014). Development of nitric oxide synthase inhibitors for neurodegeneration and neuropathic pain. *Chem. Soc. Rev.* 43 6814–6838. 10.1039/c3cs60467e 24549364 PMC4138306

[B65] Nakazawa H. Chang K. Shinozaki S. Yasukawa T. Ishimaru K. Yasuhara S. (2017). iNOS as a driver of inflammation and apoptosis in mouse skeletal muscle after burn injury: possible involvement of sirt1 s-nitrosylation-mediated acetylation of p65 NF-κB and p53. *PLoS One* 12:e0170391. 10.1371/journal.pone.0170391 28099528 PMC5242494

[B66] Nath S. Nath C. A. Pettersson K. (2008). Percutaneous lumbar zygapophysial (Facet) joint neurotomy using radiofrequency current, in the management of chronic low back pain: a randomized double-blind trial. *Spine* 33 1291–1297. 10.1097/brs.0b013e31817329f0 18496338

[B67] Ng B. K. W. Cameron A. J. D. (2010). The role of methylene blue in serotonin syndrome: a systematic review. *Psychosomatics* 51 194–200. 10.1016/s0033-3182(10)70685-x 20484716

[B68] Ng B. K. W. Cameron A. J. D. Liang R. Rahman H. (2008). Serotonin syndrome following methylene blue infusion during parathyroidectomy: a case report and literature review. *Can. J. Anesth.* 55 36–41. 10.1007/bf03017595 18166746

[B69] North R. A. (2004). P2X3 receptors and peripheral pain mechanisms. *J. Physiol.* 554 301–308. 12832496 10.1113/jphysiol.2003.048587 PMC1664762

[B70] Osmanlıoğlu H. Ø Yıldırım M. K. Akyuva Y. Yıldızhan K. Nazıroğlu M. (2020). Morphine induces apoptosis, inflammation, and mitochondrial oxidative stress via activation of TRPM2 Channel and nitric oxide signaling pathways in the hippocampus. *Mol. Neurobiol.* 57 3376–3389. 10.1007/s12035-020-01975-6 32524520

[B71] Pall M. L. (2002). NMDA sensitization and stimulation by peroxynitrite, nitric oxide, and organic solvents as the mechanism of chemical sensitivity in multiple chemical sensitivity. *Faseb J.* 16 1407–1417. 10.1096/fj.01-0861hyp 12205032

[B72] Pan P. F. Wang Y. Li X. P. Yang C. B. Zhong H. Du X. X. (2019). Effects of methylene blue on the nitric oxide-soluble guanylate cyclase-cyclic guanylyl monophosphate pathway and cytokine levels in rats with sepsis. *Int. J. Clin. Exp. Med.* 12 12203–12211.

[B73] Peng B. Pang X. Wu Y. Zhao C. Song X. (2010). A randomized placebo-controlled trial of intradiscal methylene blue injection for the treatment of chronic discogenic low back pain. *Pain* 149 124–129. 10.1016/j.pain.2010.01.021 20167430

[B74] Peng B. Zhang Y. Hou S. Wu W. Fu X. (2007). Intradiscal methylene blue injection for the treatment of chronic discogenic low back pain. *Eur. Spine J.* 16 33–38. 10.1007/s00586-006-0076-1 16496191 PMC2198898

[B75] Peter C. Hongwan D. Küpfer A. Lauterburg B. H. (2000). Pharmacokinetics and organ distribution of intravenous and oral methylene blue. *Eur. J. Clin. Pharmacol.* 56 247–250. 10.1007/s002280000124 10952480

[B76] Pollack G. Pollack A. Delfiner J. Fernandez J. (2009). Parathyroid surgery and methylene blue: a review with guidelines for safe intraoperative use. *Laryngosc.* 119 1941–1946. 10.1002/lary.20581 19598213

[B77] Polydefkis M. Hauer P. Sheth S. Sirdofsky M. Griffin J. W. Mcarthur J. C. (2004). The time course of epidermal nerve fibre regeneration: studies in normal controls and in people with diabetes, with and without neuropathy. *Brain* 127 1606–1615. 10.1093/brain/awh175 15128618

[B78] Quirion B. Bergeron F. Blais V. Gendron L. (2020). The delta-opioid receptor; a target for the treatment of pain. *Front. Mol. Neurosci.* 13:52. 10.3389/fnmol.2020.00052 32431594 PMC7214757

[B79] Rathinam V. A. Fitzgerald K. A. (2016). Inflammasome complexes: emerging mechanisms and effector functions. *Cell* 165 792–800. 10.1016/j.cell.2016.03.046 27153493 PMC5503689

[B80] Rey-Funes M. Larrayoz I. M. Fernández J. C. Contartese D. S. Rolón F. Inserra P. I. F. (2016). Methylene blue prevents retinal damage in an experimental model of ischemic proliferative retinopathy. *Am. J. Physiol. Regul. Integr. Comp. Physiol.* 310 R1011–R1019. 26984891 10.1152/ajpregu.00266.2015

[B81] Roca-Lapirot O. Fossat P. Ma S. Egron K. Trigilio G. López-González M. J. (2019). Acquisition of analgesic properties by the cholecystokinin (CCK)/CCK2 receptor system within the amygdala in a persistent inflammatory pain condition. *Pain* 160 345–357. 10.1097/j.pain.0000000000001408 30281531

[B82] Rocha P. A. Ferreira A. F. B. Da Silva J. T. Alves A. S. Martins D. O. Britto L. R. G. (2020). Effects of selective inhibition of nNOS and iNOS on neuropathic pain in rats. *Mol. Cell. Neurosci.* 105:103497. 10.1016/j.mcn.2020.103497 32353527

[B83] Roldan C. J. Nouri K. Chai T. Huh B. (2017). Methylene blue for the treatment of intractable pain associated with oral mucositis. *Pain Pract.* 17 1115–1121. 10.1111/papr.12566 28226414

[B84] Rowley M. Riutort K. Shapiro D. Casler J. Festic E. Freeman W. D. (2009). Methylene blue-associated serotonin syndrome: a ‘Green’ encephalopathy after parathyroidectomy. *Neurocrit. Care* 11 88–93. 10.1007/s12028-009-9206-z 19263250

[B85] Rygick A. N. (1968). *Atlas of the Operations on the Rectum and Colon.* Moscow: Meduch Posovie.

[B86] Saia R. S. Ribeiro A. B. Giusti H. (2020). Cholecystokinin modulates the mucosal inflammatory response and prevents the lipopolysaccharide-induced intestinal epithelial barrier dysfunction. *Shock* 53 242–251. 10.1097/shk.0000000000001355 30998649

[B87] Schiltenwolf M. Fischer C. Kunz P. (2011). How perfect studies may be? Comment on Peng et al. A randomized placebo-controlled trial of intradiscal methylene blue injection for the treatment of chronic discogenic low back pain. Pain 2010;149:124-9. *Pain* 152:954. author reply 954-955, 10.1016/j.pain.2011.01.008 21292395

[B88] Schirmer R. H. Coulibaly B. Stich A. Scheiwein M. Merkle H. Eubel J. (2003). Methylene blue as an antimalarial agent. *Redox Rep.* 8 272–275. 10.1179/135100003225002899 14962363

[B89] Schirmer R. H. Adler H. Pickhardt M. Mandelkow E. (2011). Lest we forget you–methylene blue. *Neurobiol. Aging* 32:2325.e7–16. 10.1016/j.neurobiolaging.2010.12.012 21316815

[B90] Schmid R. Evans R. J. (2019). ATP-Gated P2X receptor channels: molecular insights into functional roles. *Annu. Rev. Physiol.* 81 43–62. 10.1146/annurev-physiol-020518-114259 30354932

[B91] Schwiebert C. Irving C. Gillman P. K. (2009). Small doses of methylene blue, previously considered safe, can precipitate serotonin toxicity. *Anaesthesia* 64 924–924. 10.1111/j.1365-2044.2009.06029.x 19604213

[B92] Shanmugam G. Kent B. Alsaiwadi T. Baskett R. (2008). Serotonin syndrome following cardiac surgery. *Interact. Cardiovasc. Thorac. Surg.* 7 656–657. 10.1510/icvts.2007.173104 18334520

[B93] Starkus J. G. Heggeness S. T. Rayner M. D. (1984). Kinetic analysis of sodium channel block by internal methylene blue in pronased crayfish giant axons. *Biophys. J.* 46 205–218. 10.1016/s0006-3495(84)84014-x 6089923 PMC1435045

[B94] Starkus J. G. Rayner M. D. Fleig A. Ruben P. C. (1993). Fast and slow inactivation of sodium channels: effects of photodynamic modification by methylene blue. *Biophys. J.* 65 715–726. 10.1016/s0006-3495(93)81098-1 8218899 PMC1225774

[B95] Stephan G. Huang L. Tang Y. Vilotti S. Fabbretti E. Yu Y. (2018). The ASIC3/P2X3 cognate receptor is a pain-relevant and ligand-gated cationic channel. *Nat. Commun.* 9:1354. 10.1038/s41467-018-03728-5 PMC5893604 29636447

[B96] Sun J. Chen S.-R. Pan H.-L. (2020). μ-Opioid receptors in primary sensory neurons are involved in supraspinal opioid analgesia. *Brain Res.* 1729:146623. 10.1016/j.brainres.2019.146623 31881186 PMC6946609

[B97] Sung Y.-J. Sofoluke N. Nkamany M. Deng S. Xie Y. Greenwood J. (2017). A novel inhibitor of active protein kinase G attenuates chronic inflammatory and osteoarthritic pain. *Pain* 158 822–832. 10.1097/j.pain.0000000000000832 28059868 PMC5402717

[B98] Surprenant A. North R. A. (2009). Signaling at purinergic P2X receptors. *Annu. Rev. Physiol.* 71 333–359. 10.1146/annurev.physiol.70.113006.100630 18851707

[B99] Swanson K. V. Deng M. Ting J. P. (2019). The NLRP3 inflammasome: molecular activation and regulation to therapeutics. *Nat. Rev. Immunol.* 19 477–489. 10.1038/s41577-019-0165-0 31036962 PMC7807242

[B100] Tanga F. Y. Nutile-Mcmenemy N. Deleo J. A. (2005). The CNS role of Toll-like receptor 4 in innate neuroimmunity and painful neuropathy. *Proc. Natl. Acad. Sci. U.S.A.* 102 5856–5861. 10.1073/pnas.0501634102 15809417 PMC556308

[B101] Top W. M. Gillman P. K. De Langen C. J. Kooy A. (2014). Fatal methylene blue associated serotonin toxicity. *Neth. J. Med.* 72 179–181. 24846936

[B102] Torres-López J. E. Juárez-Rojop I. E. Granados-Soto V. Diaz-Zagoya J. C. Flores-Murrieta F. J. Ortíz-López J. U. (2007). Peripheral participation of cholecystokinin in the morphine-induced peripheral antinociceptive effect in non-diabetic and diabetic rats. *Neuropharmacology* 52 788–795. 10.1016/j.neuropharm.2006.09.015 17157334

[B103] Vutskits L. Briner A. Klauser P. Gascon E. Dayer A. G. Kiss J. Z. (2008). Adverse effects of methylene blue on the central nervous system. *Anesthesiology* 108 684–692. 10.1097/aln.0b013e3181684be4 18362601

[B104] Wainwright M. Crossley K. B. (2002). Methylene Blue–a therapeutic dye for all seasons? *J. Chemother.* 14 431–443. 10.1179/joc.2002.14.5.431 12462423

[B105] Wang F. Ma S.-B. Tian Z.-C. Cui Y.-T. Cong X.-Y. Wu W.-B. (2021). Nociceptor-localized cGMP-dependent protein kinase I is a critical generator for central sensitization and neuropathic pain. *PAIN* 162 135–151. 10.1097/j.pain.0000000000002013 32773598

[B106] Wang J. Ou S.-W. Wang Y.-J. (2017). Distribution and function of voltage-gated sodium channels in the nervous system. *Channels* 11 534–554. 10.1080/19336950.2017.1380758 28922053 PMC5786190

[B107] Wang R. Ma C. Han Y. Tan M. Lu L. (2019). Effectiveness of denervation therapy on pain and joint function for patients with refractory knee osteoarthritis: a systematic review and meta-analysis. *Pain Physician* 22 341–352. 10.36076/ppj/2019.22.341 31337163

[B108] Wang X. Zhang S. Xie Z. Chen L. Yang B. Wu X. (2019). deleterious effects of methylene blue on rat nucleus pulposus cell in vitro: changes in cell viability and secretory phenotype in exposed cells. *J. Neurol. Surg. A Cent. Eur. Neurosurg.* 80 174–179. 10.1055/s-0038-1670638 30818407

[B109] Wolin M. S. Cherry P. D. Rodenburg J. M. Messina E. J. Kaley G. (1990). Methylene blue inhibits vasodilation of skeletal muscle arterioles to acetylcholine and nitric oxide via the extracellular generation of superoxide anion. *J. Pharmacol. Exp. Ther.* 254 872–876. 2168487

[B110] Wolvetang T. Janse R. Ter Horst M. (2016). Serotonin syndrome after methylene blue administration during cardiac surgery: a case report and review. *J. Cardiothorac. Vasc. Anesth.* 30 1042–1045. 10.1053/j.jvca.2015.11.019 27130452

[B111] Yadav S. Surolia A. (2019). Lysozyme elicits pain during nerve injury by neuronal Toll-like receptor 4 activation and has therapeutic potential in neuropathic pain. *Sci. Transl. Med.* 11:eaav4176. 10.1126/scitranslmed.aav4176 31391320

[B112] Yang Y. Li Q. He Q.-H. Han J.-S. Su L. Wan Y. (2018). Heteromerization of μ-opioid receptor and cholecystokinin B receptor through the third transmembrane domain of the μ-opioid receptor contributes to the anti-opioid effects of cholecystokinin octapeptide. *Exp. Mol. Med.* 50 1–16. 10.1038/s12276-018-0090-5 29780163 PMC5960647

[B113] Yang Y. Wang H. Kouadir M. Song H. Shi F. (2019). Recent advances in the mechanisms of NLRP3 inflammasome activation and its inhibitors. *Cell Death Dis.* 10:128. 10.1038/s41419-019-1413-8 PMC6372664 30755589

[B114] Ye Y. Wang Y. Yang Y. Tao L. (2020). Aloperine suppresses LPS-induced macrophage activation through inhibiting the TLR4/NF-κB pathway. *Inflammation Res.* 69 375–383. 10.1007/s00011-019-01313-0 32144444

[B115] Yeung A. W. K. Georgieva M. G. Atanasov A. G. Tzvetkov N. T. (2019). Monoamine oxidases (MAOs) as privileged molecular targets in neuroscience: research literature analysis. *Front. Mol. Neurosci.* 12:143. 10.3389/fnmol.2019.00143 31191248 PMC6549493

[B116] Zhang H. Li F. Li W. W. Stary C. Clark J. D. Xu S. (2016). The inflammasome as a target for pain therapy. *Br. J. Anaesth.* 117 693–707. 10.1093/bja/aew376 27956668 PMC5155560

[B117] Zhang X. Hao J. Hu Z. Yang H. (2016). Clinical evaluation and magnetic resonance imaging assessment of intradiscal methylene blue injection for the treatment of discogenic low back pain. *Pain Physician* 19 E1189–E1195. 27906950

[B118] Zhang L. Liu Y. Huang Z. Nan L. Wang F. Zhou S. (2019). Toxicity effects of methylene blue on rat intervertebral disc annulus fibrosus cells. *Pain Physician* 22 155–164. 10.36076/ppj/2019.22.155 30921981

[B119] Zhang Y. Zhao J. Zhang T. Yang Z. (2010). In vitro assessment of the effect of methylene blue on voltage-gated sodium channels and action potentials in rat hippocampal CA1 pyramidal neurons. *Neurotoxicology* 31 724–729. 10.1016/j.neuro.2010.07.001 20621122

[B120] Zhao X. Tang Z. Zhang H. Atianjoh F. E. Zhao J.-Y. Liang L. (2013). A long noncoding RNA contributes to neuropathic pain by silencing Kcna2 in primary afferent neurons. *Nat. Neurosci.* 16 1024–1031. 10.1038/nn.3438 23792947 PMC3742386

[B121] Zheng J. Li Q. (2019). Methylene blue regulates inflammatory response in osteoarthritis by noncoding long chain RNA CILinc02. *J. Cell Biochem.* 120 3331–3338. 10.1002/jcb.27602 30548658

[B122] Zuschlag Z. D. Warren M. W. Schultz S. K. (2018). Serotonin toxicity and urinary analgesics: a case report and systematic literature review of methylene blue-induced serotonin syndrome. *Psychosomatics* 59 539–546. 10.1016/j.psym.2018.06.012 30104021

